# Prevalence and Features of Incidental Findings in Veterinary Computed Tomography: A Single-Center Six-Years’ Experience

**DOI:** 10.3390/ani13040591

**Published:** 2023-02-08

**Authors:** Tiziana Caspanello, Marisa Masucci, Diego Iannelli, Nicola Maria Iannelli, Massimo De Majo

**Affiliations:** 1 Department of Veterinary Science, University of Messina, 98168 Messina, Italy; 2 Clinica Veterinaria Camagna–VetPartners, 89124 Reggio di Calabria, Italy

**Keywords:** incidentaloma, computed tomography, imaging, dog, cat

## Abstract

**Simple Summary:**

Through advanced diagnostic imaging such as computed tomography (CT), clinicians can obtain a diagnosis more easily, but they also may find unexpected imaging findings in different organs and tissues called “incidentalomas”. Veterinarians do not have clear evidence-based indications about what an incidentaloma is and how to manage incidentalomas, thereby increasing the risk of unnecessary investigations. This retrospective study aims to investigate incidentalomas in CT exams carried out over six years in a veterinary facility, and to describe the prevalence, sites, types of incidentalomas and their follow-up, their correlations or associations with the species, breed, sex, and age of the patients examined and with the anatomic location. Five hundred and sixty-one CT exams performed on 512 dogs and 49 cats were evaluated. There were 80 incidentalomas in 57 dogs and four cats. In dogs, the incidentalomas occurred particularly in Boxers and older animals, and more frequently in neck, thoracic, and abdominal scans. Spinal incidentalomas were the most common typologies in dogs. This study may allow for the growth of awareness about incidentalomas and can help veterinary clinicians set up the evidence-based clinical management of incidental findings.

**Abstract:**

Computed tomography (CT) is an advanced imaging technique that may lead to detect “incidentalomas”, unexpected asymptomatic lesions found during unrelated examinations. Their clinical meaning and management are not clear for veterinarians, who risk unnecessary investigations that harm the patients. This study is a retrospective analysis that aims to investigate incidentalomas in CT exams and to describe their prevalence, location, types and follow-up, their correlations and associations with the species, breed, sex, and age of patients examined and with the kind and number of sites scanned. The reports of 561 CT scans performed in 512 dogs and 49 cats in a veterinary facility over six years were reviewed and compared to the clinical records of the patients. Eighty incidentalomas were found in 57 dogs and four cats. A significant positive correlation was found in dogs between age and the prevalence of incidentalomas. In dogs, the prevalence of incidentalomas was significantly higher in Boxers and in neck, thoracic, and abdominal scans. Spinal incidentalomas were the most common typologies in dogs. This study can represent a tool that allows clinicians to acquire greater awareness about incidentalomas and to carry out the evidence-based clinical management of them.

## 1. Introduction

The improvement in imaging techniques such as computed tomography (CT) over the last several years has led to the routine identification of organs and lesions that were previously unattainable with the available technology [1], allowing for the better detection of diseases and enabling clinicians to make correct decisions about their patient’s treatment [2]. However, advances in diagnostic imaging have also created a dilemma for clinicians and radiologists regarding the possibility of the accidental detection of asymptomatic lesions in different organs, called “incidentalomas” [3].

Gesbert et al. analyzed the evolution of the meaning of the term “incidentaloma” from the 1980s to the present [4]. The term was born in 1982 to define the increasingly frequent discovery of clinically asymptomatic masses in the adrenal glands, which were only recently observable, thanks to new emerging imaging techniques [5,6,7]. Only in the early 2000s did this term appear as a “keyword” for the indexing of radiology, surgery, and endocrinology medical-scientific publications, with substantial uncertainties about the meaning and the usefulness of this neologism [8]. Initially restricted to the field of endocrinology, referring to masses found on the thyroid [9] or pituitary gland [3], the term “incidentaloma” was then extended to the liver, heart, uterine appendages, small intestine, etc. [10,11,12,13], being applied to any unexpected tumor discovered by imaging, whose meaning was indeterminate. Ten years later, the American College of Radiology (ACR) developed recommendations to manage these unexpected discoveries and proposed a consensus definition of incidentaloma as “an incidentally discovered mass or lesion, detected by CT, MRI, or other imaging modality performed for an unrelated reason” [14]. Subsequently, fields of medicine such as genomics have acquired the term and re-evaluated its meaning; from 2013 and 2017, in fact, incidental findings were redefined as “secondary”, because when evaluating an area, in some way, the operator searches for any possible alterations that may be involved, which is guided, but not limited, to clinical indications. Thus, incidentalomas may no longer be serendipitous or unexpected, but secondary to examination [4,15,16,17].

In diagnostic imaging, the term incidentaloma still refers to an unexpected, asymptomatic lesion discovered by chance during the investigation of an unrelated condition and it represents one of the drawbacks of using more sensitive diagnostic tests [2,18,19,20,21].

In human medicine, it is reported that the greatest number of incidental findings was observed in thoracic CT (45%), and most of the malignant incidentalomas were localized to the breast (42%) and ovary (28%), and prostatic and colic incidentalomas represented, respectively, 11% and 17% of the incidental findings, resulting in being malignant in 10–20% of cases, while cerebral and adrenal incidentalomas had remarkably low rates of malignancy [22].

Veterinary tomographic incidental findings have been reported in studies dealing with incidentalomas or subclinical findings in certain anatomical sites (e.g., middle ear lesions, adrenal nodules, thyroid tumors, and anal sacculiths [23,24,25,26]), and works that describe various pathologies in certain organs or regions (e.g., migrating intrathoracic grass awns, prostatic alterations, ear disease, thoracic abnormalities, hyoid fractures, retrobulbar diseases [20,27,28,29,30,31]), among which anomalies found accidentally are also mentioned.

In both human and veterinary medicine, there is a concerning issue about the difficulty of the clinician in recognizing and distinguishing a potentially dangerous incidental finding that needs further investigation and treatment from a harmless and negligible incidentaloma. Additionally, incidentalomas increase the risk of overdiagnosis, represented by unnecessary investigations that involve more disadvantages than benefits to the patients, both from a medical and an economic point of view [2,21,22].

The aims of this study were to describe the incidentalomas found during the computed tomography investigations carried out over six years of activity at a veterinary facility to provide a scientific contribution about the prevalence, anatomical locations, the main types of incidental findings and follow-up, and their association or correlation with the species, breed, sex, and age of patients examined and with the kind and number of sites was also evaluated.

## 2. Materials and Methods

The reports of all CT exams performed between August 2015 (25 August 2015) and November 2021 (9 November 2021) at a private veterinary clinic (Clinica Veterinaria Camagna, Reggio di Calabria, Calabria, Italy, 38°06′54″ N, 15°39′25″ E) were examined and compared to the clinical records of the patients. All images were acquired using a Revolution act 16-layer CT scanner (GE Healthcare, Chicago, IL, USA). After the acquisition, the images were post-processed based on the request for the case; then, both the raw images and post-produced ones, along with the indication for the CT and a brief resume of the history of the patients, were sent to a reporting center for interpretation by a team of five graduates of the European College of Veterinary Diagnostic Imaging. The information on the signalment, a brief medical history, the indication for the exam, the description, and the comment of the specialist were included in the report.

**Scheme 1:**
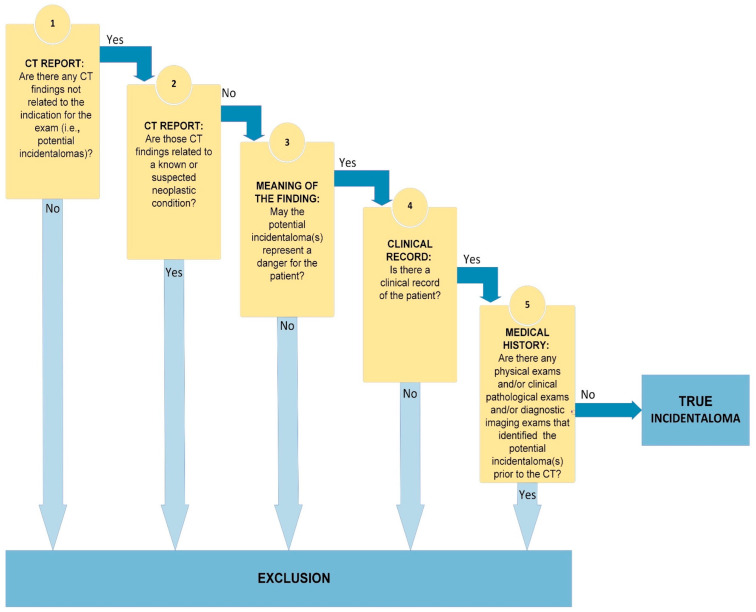
Decision algorithm for the inclusion or exclusion of tomographic findings as incidentalomas.

The criteria of exclusion were: (i) negative exams; (ii) exams fully matching the indication and/or the clinical records of the patients (i.e., physical exams, clinical pathological exams, radiography and ultrasound exams); (iii) follow-up CT exams that did not show any different findings than the previous ones; (iv) incidental findings representing congenital anatomical anomalies that did not represent a danger to the health and life of the patient (e.g., double vena cava, transitional vertebrae, accessory spleen); (v) acquired incidental findings that would not become harmful or clinically evident (e.g., single renal cysts, fat accumulations in corpora cavernosa); (vi) incidental findings potentially secondary to neoplasms (possible metastases, reactive lesions, etc.) found in CT exams performed for known or suspected neoplastic conditions. All of the remaining CT findings not mentioned in the clinical records nor matching the indication of the CT exam were included and considered as incidentalomas (Scheme 1). Follow-up data of the patients included were also collected.

### Statistical Analysis

Statistical analysis was performed using GraphPad Prism version 7.0 for Windows (GraphPad Software, San Diego, CA, USA). The distribution of continuous variables was evaluated by the D’Agostino–Pearson omnibus normality test.

Fisher’s exact test was used to evaluate associations between the presence of incidentalomas and the species, breed (for dogs only), sex, and site examined. Data with 0 frequency were excluded from Fisher’s exact test. The Spearman’s rank correlation test was used to assess the correlation between the prevalence of incidentalomas and age. The Pearson’s correlation test was used to evaluate the correlation between the prevalence of incidentalomas and the number of sites examined. *p* values < 0.05 were considered significant.

## 3. Results

### 3.1. Incidental Findings

Five hundred and sixty-one CT exams performed on 512 dogs and 49 cats were evaluated. Sixty-one exams performed on 57 dogs (29 males; 28 females) and four cats (two males; two female) met the inclusion criteria and were considered as presenting incidentalomas. CT exams showed a total of 80 incidentalomas (76 in dogs, four in cats) (Table 1). Fifteen out of 57 (26.32%) dogs showed more than one incidentaloma, four (7.02%) had three incidentalomas each, and 11 (19.3%) had two incidentalomas each. Each cat included had one incidentaloma.

### 3.2. Incidental Findings

**Scheme 2:**
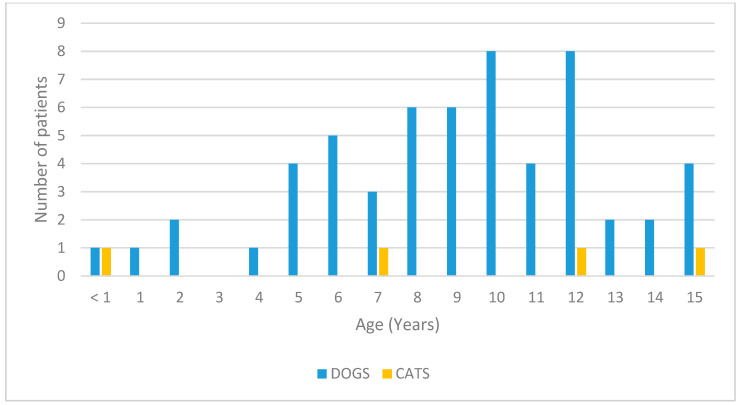
Age of the dogs and cats with incidentaloma.

Signalment data are shown in Table 2. Dogs were aged between 2 and 204 months (median = 84 months, 25th percentile = 48 months, 75th percentile = 120 months). Cats were aged between 3 and 204 months (median = 84 months, 25th percentile = 12 months, 75th percentile = 144 months). The mean age of dogs and cats with incidentalomas was 111.1 (SD = 47.73) and 104 (SD = 75.26) months, respectively, with dogs aging between 10 and 276 months, and cats between 8 and 180 months (Scheme 2). A significant positive correlation was found in dogs between age and prevalence of incidentalomas (*p* = 0.0001; r = 0.6218). The purebred dogs belonged to 56 different breeds. The breeds of dogs presenting incidentalomas are shown in Table 3. The breeds showing the highest average number of incidentalomas were French Bulldog (*n* = 2), Cavalier King Charles Spaniel (*n* = 2), Greyhound (*n* = 2), Schnauzer (*n* = 2), Labrador Retrievers (*n* = 1.67), and Boxers (*n* = 1.5). Prevalence of incidentalomas in Boxers (6/7 = 85.71%) was significantly higher than in the mixed breed dogs (17/160 = 10.63%) (*p* < 0.0001; OR = 50.47; 95% CI = 7.067–580.9). Table 3 shows the absolute and average number of incidentalomas found in the patients included.

### 3.3. Sites Examined

**Figure 1:**
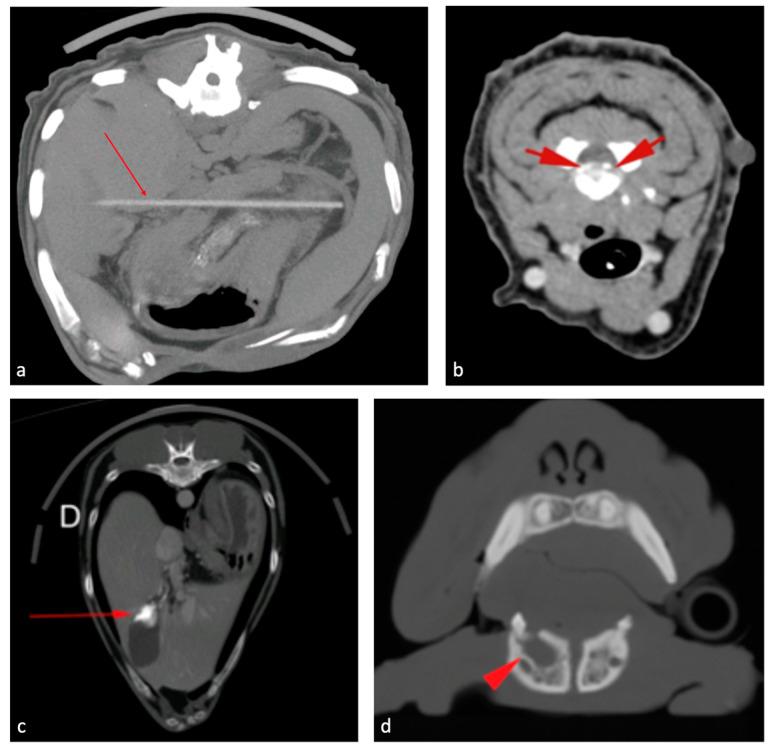
Examples of incidentalomas found in the CT examinations. (**a**) Peritoneal foreign body (arrow); (**b**) intervertebral C3/C4 disc herniation (arrows); (**c**) cholelithiasis (arrow); (**d**) mandibular cyst (arrow tip).

The number and prevalence of incidentalomas at the different sites examined in dogs and cats are shown in Table 4 and Table 5, respectively. In dogs, the prevalence of incidentalomas found in the neck was significantly higher than in the cervical vertebral segment (*p* = 0.0216; OR = 2.801; 95% CI = 1.222–6.404), thoracic vertebral segment (*p* = 0.0273; OR = 2.509; 95% CI = 1.101–5.533), lumbar vertebral segment (*p* = 0.0147; OR = 2.809; 95% CI = 1.189–6.816), and sacral vertebral segment (*p* = 0.0392; OR = 2.532; 95% CI = 1.068 to 6.166) (Table 4). Moreover, the prevalence of incidentalomas detected in the thorax was significantly higher than in the cervical vertebral segment (*p* = 0.0015; OR = 3.397; 95% CI = 1.574 to 7.220), thoracic vertebral segment (*p* = 0.0021; OR = 3.043; 95% CI = 1.460–6.162), lumbar vertebral segment (*p* = 0.0010; OR = 3.406; 95% CI = 1.571–7.354), and sacral vertebral segment (*p* = 0.0029; OR = 3.071; 95% CI = 1.410 to 6.653) (Table 4). Finally, the prevalence of incidentalomas found in the abdomen was significantly higher than in the head (*p* = 0.0155; OR = 1.966; 95% CI = 1.128–3.364), thoracic vertebral segment (*p* = 0.0006; OR = 3.396; 95% CI = 1.678–6.871), lumbar vertebral segment (*p* = 0.0002; OR = 3.802; 95% CI = 1.806–8.073), and sacral vertebral segment (*p* = 0.0009; OR = 3.428; 95% CI = 1.620–7.304) (Table 4). The number and prevalence of incidentalomas depending on the number of sites examined in dogs and cats are shown in Table 6 and Table 7, respectively. The typologies and number of incidentalomas in dogs and cats are shown in Table 8. Table 9 shows the typologies of the incidental findings based on the age of the dogs and cats. Figure 1 shows some examples of the incidentalomas found by CT. 

### 3.4. Follow-Up

Thirty-two out of 61 patients with incidentalomas died or were lost to follow-up. Of the 29 remaining, 14 came back for treatment or pursuit of the initial conditions that led to the CT exam; 12 came back for other reasons, not related to the indication of the CT and/or the incidentalomas; two patients had the incidentalomas followed-up (i.e., prostatic cysts, adenopathy) and one went to surgery (i.e., foreign body).

## 4. Discussion

### 4.1. Incidental Findings: Prevalence and Features

Incidentalomas, unexpected findings of potential clinical significance, may be observed during imaging tests aimed at other suspects [2,14]. Veterinarians who use advanced imaging techniques such as CT may not have clear evidence-based indications on: (a) what an incidental finding is; (b) which types occur most often and how often; and (c) which incidental findings are worthy of further diagnostic and/or therapy and which, on the other hand, are irrelevant from a clinical point of view [2,21,32]. The present study represents an investigation on the incidental findings in computed tomography in dogs and cats; it is not limited to a single site or pathology, but it describes all the possible incidentalomas that can be found in clinical practice. Incidentalomas occurred in 11.13% of dogs and 8.16% of cats that presented at least one incidentaloma and 19.73% of dogs had more than one. Despite the expectation that the more extensive the area explored and the older the patient that there was a greater likelihood of seeing an incidental finding [4], our results only partially fit this statement. In fact, in dogs, there was a significant positive correlation between the age and prevalence of incidentalomas, and Boxers had a significantly higher prevalence of incidentalomas, but there was no positive correlation between the number of sites examined and the prevalence of incidental findings. The former may be the consequence of a higher prevalence of comorbidities and chronic/degenerative diseases in older dogs [33]. The second may be related to the fact that Boxers carry many predispositions to disease, some of which have been found in our study as incidentalomas (e.g., mandibular cysts [34], otitis [35]), although there may be other unknown reasons. Therefore, when performing a CT scan on a Boxer dog and/or an old dog, there is a higher probability of an incidental finding, regardless of how extended the field of examination is.

Types of tomographic incidentalomas reported in the literature are migrant thoracic grass awns [27], prostatic cysts and heterogeneity [28], middle ear lesions [23,29], adrenal masses [24], thoracic abnormalities [20], thyroid lesions [25], hyoid fractures [30], retrobulbar diseases [31], and anal sacculiths [26]. The most common types of incidental findings pointed out in this research were spinal diseases (most of all, intervertebral disc herniation); although a portion of the spine is included in almost all scans (i.e., neck, chest, abdomen, total body), the prevalence of incidentalomas in the spine was significantly lower than in the neck, thoracic, and abdominal scans, and also in the head compared to abdominal scans. The high number of incidental spinal findings may be related to the fact that radiology has a lower sensitivity than CT [2,36,37,38]. Therefore, it is easier for a lesion in these sites to escape routine X-rays and to be discovered only by CT. Furthermore, it has to be considered that X-ray scans were not performed on each patient of ours prior to the CT, therefore, we cannot assess if any of these incidentalomas would have been radiologically evident. Spinal diseases may also stay subclinical as long as they do not create a compression of the spinal cord and nerve roots. It is reported that disco-spondylitis may develop vague symptoms and sometimes they do not cause any pain, so they may be difficult to diagnose, and some lumbosacral CT abnormalities may be clinically silent, particularly in old dogs [37,39]. A similar consideration could also be applied to airway lesions; in fact, since CT is the imaging technique of choice for the chest [40], the result obtained regarding the prevalence of chest lesions probably reflects the greater sensitivity of computed tomography in imaging this district [2,21,41]. On the other hand, the prevalence of incidentalomas in the limbs may have been underreported, both because limbs were only included in specific scans and total-body CT scans and, in general, this site has been less investigated. In our study, the prevalence of middle ear incidentalomas was lower than that previously reported in CT scans [23,29], since they were only noted in 1/24 (4.17%) and 5/206 (2.43%) scans of the head performed in cats and dogs, respectively. With regard to incidental adrenal masses, in our study, they appeared to have a much lower prevalence (0.98%) than those reported in dogs [24]. However, this discrepancy could be the consequence of the different methods adopted, in fact, in previous studies, less restrictive inclusion criteria were used, admitting patients undergoing CT for a suspected neoplasm which, conversely, were excluded from our study. Furthermore, in previous studies, the authors also included six incidentalomas found both by ultrasound and by CT in their results, while these types of cases were excluded from our study. Similarly to Bertolini et al. [25], in our study the incidence of thyroid nodules in the neck CT of dogs was 0.89%, while in cats, there was one in 20 neck scans (5%). Subclinical CT thoracic abnormalities reported by other authors in cats were: atelectasis, bronchial lesions, space occupying lesions, pulmonary nodules, other, ground-glass opacity, consolidation, and thoracic wall lesions [20]. In our study, both in dogs and cats, the thoracic lesions included were: bronchial collapses, bronchopneumonia, pulmonary thickening, hiatal hernia, and a migrating foreign body. Innocuous thoracic lesions such as small air-trappings were excluded from our study because of their clinical irrelevance. Although nonrelated to computed tomography, it has been reported that 9/16 and 3/27 patients affected by hiatal hernia did not show any clinical signs [42,43]. In the former study, three cases were radiographic incidental findings, two cases were found during necropsy, and the remainder were evaluated by X-rays; in the latter, hiatal hernia was diagnosed by contrast radiography of the esophagus, fluoroscopy, or esophagoscopy. Of the other types of CT incidental findings described in the literature such as hyoid bone fractures in dogs and cats [30], retrobulbar pathologies in dogs [31], and anal sac lithiasis in dogs [26], no findings occurred in our study. Finally, with regard to the remaining types of incidental CT findings resulting from our study (i.e., renal, hepatic, oral, splenic, pituitary, articular, cardiovascular, pancreatic incidentalomas, incidental urolithiasis, foreign bodies, abscesses, and adenopathies), to our knowledge, there are no references in the literature for a comparison.

### 4.2. Incidental Findings and Follow-Up

Results of the follow-up show that after the discovery of the incidentalomas, only three patients had them followed-up or treated and the greatest part of follow-ups regarded the primary reasons that made them get a CT exam. Most of the patients were lost to follow-up or died. Considering that some of the CT exams performed in the clinic were requested from other structures, it is likely that part of these patients was followed-up in those structures. While some authors have stated that when facing a new finding there is the tendency to put aside the primary condition [2], our results show that clinicians focus on solving the main problems of the animal, which cause an illness that must be investigated and resolved. Therefore, with few exceptions, the incidental findings were of lower priority, perhaps because they were still non-pathological or non-symptomatic. In both human and veterinary medicine, there is a concerning issue about the clinician’s difficulty in recognizing and distinguishing a potentially dangerous incidental finding that needs further investigations and treatment from a harmless and negligible incidentaloma.

### 4.3. Incidental Findings and Overdiagnosis

Incidentalomas also increase the risk of unnecessary investigations that involve more disadvantages than benefits to the patients, both from a medical and an economic point of view [2,20,21]. The growing use of advanced diagnostic technologies may facilitate overdiagnosis [44,45]. This problem is defined as the correct identification of a disease or a lesion that will never cause clinical signs or clinical harm, and if pursued with further tests and/or treatments, will cause patients more harm than good [32]. Treating an over-diagnosed condition provides no benefits but can cause physical and psychological harm and generate costs [2,46]. While increasingly sensitive diagnostic technologies allow the detection of many potentially severe chronic diseases at the earliest stages, they may also expand the disease reservoir of subclinical conditions that generate overdiagnosis [44]. To avoid the risk of overdiagnosis, we limited our inclusion criteria to those incidental findings that should warn the clinician as being more likely to evolve into conditions that threaten the patient’s health. In fact, incidental findings are not automatically overdiagnoses, but, if clinically irrelevant, they are among the factors that may facilitate overdiagnosis as well as the broadening of disease definitions (lowering of diagnostic thresholds and recognition of risk factors as pre-diseases); the use of advanced technology for diagnosis and the use of more sensitive screening tests; the widespread screening programs offered by public health interventions; cultural factors (the value of diagnosis for its own sake); system factors (financial incentives for more testing); and the lack of evidence regarding disease spectrum in studies of diagnostic accuracy [32,44,45]. In human medicine, the problem of overdiagnosis has been extensively investigated and it has been present in some form since before the 20th century, but it has become particularly acute since the early 21st century [47], and nowadays, it is a growing issue [44]. Starting from 2012, an awareness campaign for clinicians [48] and a society aimed at spreading knowledge about overdiagnosis and prevention strategies for this problem [49] were founded. In veterinary medicine, the risks of overdiagnosis associated with common diagnostic practices is still out of focus, and its extent in this field is still unclear, although there are some potential risk factors shared with human medicine. In fact, while a different economic model may reduce the indiscriminate use of testing and treatment because usually the clients pay directly for it, psychological factors that drive overdiagnosis in the human medical field as well as the financial interests and the expectation that screening and early disease detection always benefit the patients are also present in veterinary medicine [32]. Additionally, the growth of the pet insurance industry may facilitate overtreatment [50]; surveys of pet owners demonstrated that those with insurance are more inclined to spend more and receive more services (testing and treatment) suggested by their veterinarian because they have pet health insurance [51,52]. Moreover, the financial burden of tests and investigations that follow incidental findings is not to be underestimated, and may affect the animals’ health and survival; overdiagnosis and overtreatment reduce the affordability of care, increasing the overall care costs and waste economic resources that could otherwise be used for more worthy tests and treatments [32]. In this scenario, clinicians play a key role in choosing what is best for the patient [50]. Overtreatment that follows overdiagnosis is often caused by the absence of evidence-based treatment selection criteria [53]. Given the lack of evidence-based guidelines in veterinary medicine, we could apply helpful strategies found in human medicine such as the educating of clinicians and patients about the risk factors, a review of clinical practice guidelines to reduce the diagnostic procedures related to overdiagnosis, choosing selective and targeted screening tests and investigations, and expanding the research to identify and quantify overdiagnosis. Unexpected abnormal findings should be evaluated within the full clinical picture and need to be verified before making a diagnosis or considering treatment [32]. The value of an incidentaloma is subordinated to the seriousness of the concomitant problems and to the primary reason for which the animal was subjected to the CT scan, and focusing on the primary problem for which the patient presented is a priority [2]. On the other hand, our study showed that, although the detection of incidentalomas occurred in veterinary medicine, it did not evolve into overdefinition or overtreatment; since only potentially harmful incidentalomas were further evaluated, there seems to be, rather, a risk of “underdefinition” and “undertreatment” of potentially dangerous incidental findings, which, if not investigated, monitored and/or treated, could compromise the patient’s health.

### 4.4. Limits of the Study

The retrospective design of the study brought with it the limit of working on indirect data rather than the direct information of patients. In fact, within the structure that performed the CTs, there were several veterinary doctors who performed the visits and filled in the medical records, so the information reported was subject to a certain degree of inter-operator variability. Another important limitation concerned the veterinary clinic working as a reference center for computed tomography who sent the images to a reporting center for interpretation by a team of expert radiologists. Technical parameters used for acquisition (e.g., CT density, Window settings, etc.) played a marginal role in the incidental findings because the post-processed images were sent along with the raw images, so that the radiologist could edit them based on their judgement. Since more than one radiologist interpreted the images, inter-operator variability may have partially influenced our results. In addition, teleradiology may hinder the work of the radiologist [21] and may have increased the loss in clinical information. In fact, if the requesting center and/or the referral center sent incomplete anamnestic data (only the ones linked to the indication for the CT) to the reporting center, the radiologist would be led to consider all of the tomographic findings unrelated to the primary problem as incidental; when there were incomplete medical records, it was impossible to verify whether and which of these findings had already been diagnosed by other exams. Hence, we excluded all the cases without a medical record from the study and considered only unquestionable incidentalomas. However, when there was no medical record and we could not say that a CT finding was an incidentaloma, neither could we say that it was not; therefore, we may also have excluded some CT findings that with some probability, but without certainty, were incidentalomas. Moreover, the radiologist loses the overall vision, being able to analyze each tomographic finding only as single and separate entity, apart from their clinical context. All of this not only hindered our study, but also reflects a general obstacle in the global understanding of a clinical case and may raise considerations on how medicine by reference works.

## 5. Conclusions

This study demonstrated that incidental findings in computed tomography scans are a phenomenon present in veterinary medicine, particularly in the Boxer breed and older dogs, and with a higher prevalence in neck, thoracic, and abdominal scans. Spinal incidentalomas (in particular, disc herniations) were the most frequent typologies.

Through the analysis of the prevalence of the different types of incidental findings, compared to the characteristics of the patients, this study can represent a tool that allows clinicians to acquire a greater awareness about incidentalomas and to set up evidence-based clinical management when facing an incidental finding.

## Figures and Tables

**Table 1:** Absolute and average number of incidentalomas found in 57 dogs and four cats in the CT examinations. Since there was one incidentaloma in each cat and all cats with incidentaloma were domestic shorthair, the sex and breed in cats are not reported.

Signalment	Number of Patients Included	Number of Incidentalomas	Average Number of Incidentalomas
Species			
Dog	57	76	1.33
Cat	4	4	1
Sex (dogs)			
Male	29	43	1.48
Female	28	33	1.18
Breed (dogs)			
Mixed breed	17	25	1.47
Purebred	40	51	1.28

**Table 2:** Data for signalment [*n* (%)] of the total enrolled animals and of animals with or without incidentalomas in the CT examinations.

Signalment	Total	Presence of Incidentalomas	Absence of Incidentalomas
Species			
Dog	512 (91.27)	57 (11.13)	455 (88.87)
Cat	49 (8.73)	4 (8.16)	45 (91.84)
Sex (dogs)			
Male	257 (50.19)	29 (11.28)	228 (88.72)
Female	232 (45.31)	28 (12.07)	204 (87.93)
NK ^1^	23 (4.49)	0 (0)	23 (100)
Sex (cats)			
Male	30 (61.22)	2 (6.67)	28 (93.33)
Female	19 (38.77)	2 (10.53)	17 (89.47)
Breed (dogs)			
Mixed breed	160 (31.25)	17 (10.63)	143 (89.38)
Purebred	349 (68.16)	40 (11.46)	309 (88.54)
NK ^1^	3 (0.59)	0	3 (100)
Breed (cats)			
DSH ^2^	48 (97.96)	4 (8.33)	44 (91.67)
Persian	1 (2.04)	0 (0)	1 (100)

^1^ NK: not known; ^2^ DSH: domestic shorthair.

**Table 3:** Breeds of dogs with incidentalomas in the CT examinations.

Breed	Number of Dogs
Deutscher Boxer	6
German Shepherd	3
Labrador Retriever	3
Rottweiler	3
Cane Corso Italiano	2
Cavalier King Charles Spaniel	2
Dobermann	2
English Setter	2
American Staffordshire	1
Chihuahua	1
Chow Chow	1
Dachshund	1
English Cocker Spaniel	1
English Springer Spaniel	1
French Bulldog	1
German Spitz	1
Greyhound	1
Miniature Pinscher	1
Newfoundland	1
Pug	1
Schnauzer	1
Shar Pei	1
Shih Tzu	1
Siberian Husky	1
Yorkshire Terrier	1

**Table 4:** Sites examined in the total number of enrolled dogs and in dogs with or without incidentalomas in the CT examinations [*n* (%)].

Site Examined	Total	Presence of Incidentalomas	Absence of Incidentalomas
Head *	206 (14.88)	24 (11.65)	182 (88.35)
Neck *	112 (8.09)	18 (16.07)	94 (83.93)
Thorax *	191 (13.80)	36 (18.85)	155 (81.15)
Abdomen *	204 (14.74)	42 (20.59)	162 (79.41)
Fore limbs	41 (2.96)	6 (14.63)	35 (85.37)
Hind limbs	27 (1.95)	4 (14.81)	23 (85.19)
Spinal Segments			
Cervical *	125 (9.03)	8 (6.40)	117 (93.60)
Thoracic *	141 (10.19)	10 (7.09)	131 (92.91)
Lumbar *	141 (10.19)	9 (6.38)	132 (93.62)
Sacral *	128 (9.25)	9 (7.03)	119 (92.97)
Caudal	68 (4.91)	7 (10.29)	61 (89.71)

* = significant difference.

**Table 5:** Sites examined in the total number of enrolled cats and in cats with or without incidentalomas in the CT examinations [*n* (%)].

Site Examined	Total	Presence of Incidentalomas	Absence of Incidentalomas
Head	24 (16.00)	2 (8.33)	22 (91.67)
Neck	20 (13.33)	1 (5.00)	19 (95.00)
Thorax	33 (22.00)	2 (6.06)	31 (93.94)
Abdomen	35 (23,33)	3 (8.57)	32 (91.43)
Fore limbs	5 (3.33)	0 (0)	5 (100)
Hind limbs	4 (2.67)	0 (0)	4 (100)
Spinal Segments			
Cervical	6 (4.00)	0 (0)	6 (100)
Thoracic	6 (4.00)	0 (0)	6 (100)
Lumbar	6 (4.00)	0 (0)	6 (100)
Sacral	6 (4.00)	0 (0)	6 (100)
Caudal	5 (3.33)	0 (0)	5 (100)

**Table 6:** The number of sites examined in the total number of enrolled dogs and in dogs with or without incidentalomas in the CT examinations [*n* (%)].

Number of Sites Examined	Total	Presence of Incidentalomas	Absence of Incidentalomas
1	174 (33.98)	12 (6.89)	162 (93.10)
2	127 (24.80)	23 (18.11)	104 (81.89)
3	52 (10.16)	6 (11.54)	46 (88.46)
4	89 (17.38)	8 (8.99)	81 (91.01)
5	49 (9.57)	3 (6.12)	46 (93.88)
6	4 (0.78)	1 (25.00)	3 (75.00)
7	3 (0.59)	0 (0.00)	3 (100.00)
11	14 (2.73)	4 (28.57)	10 (71.43)

**Table 7:** The number of sites examined in the total number of enrolled cats and in cats with or without incidentalomas in the CT examinations [*n* (%)].

Number of Sites Examined	Total	Presence of Incidentalomas	Absence of Incidentalomas
1	14 (28.57)	2 (14.29)	12 (85.71)
2	13 (26.53)	1 (7.69)	12 (92.31)
3	7 (14.29)	0 (0.00)	7 (100.00)
4	10 (20.41)	1 (10.00)	9 (90.00)
5	1 (2.04)	0 (0.00)	1 (100.00)
11	4 (8.16)	0 (0.00)	4 (100.00)

**Table 8:** The number, location, type, and species in which the incidentalomas were found in the CT examinations.

Typologies	Dogs	Cats
HEAD		
EAR		
Otitis media	3	1
Otic polyp	1	
Otitis externa	1	
Otolithiasis	1	
MOUTH		
Sialocele	2	
Mandibular cyst	1	
THORAX		
AIRWAY		
Bronchial collapse	3	
Bronchopneumonia	1	1
Pulmonary thickening	1	
CARDIO-VASCULAR SYSTEM		
Pulmonary thromboembolism	1	
ABDOMEN		
GASTROINTESTINAL TRACT AND ACCESSORY GLANDS		
Hiatal hernia	4	
Cholelithiasis	2	
Pancreatic volume increase and peripancreatic steatitis	1	
PERITONEUM		
Nodule	1	
SPLEEN		
Nodule	4	
UPPER AND LOWER URINARY TRACT		
Renal lithiasis/dystrophic mineralization	3	
Urolithiasis	1	
PROSTATE		
Cysts/BPH ^1^	5	
ENDOCRINE SYSTEM		
PITUITARY GLAND		
Volume increase	1	
ADRENAL GLANDS		
Neoplasm	2	
Hyperplasia		1
THYROID		
Nodule	1	1
SPINE AND JOINTS		
Herniated disc	17	
Foraminal stenosis	4	
Spondylitis, disco-spondylitis, spondylarthrosis	3	
Facet joint hypertrophy	2	
Congenital/traumatic deviation	1	
Intracanal fat accumulation	1	
Patellar luxation	1	
OTHER		
Foreign body ^2^	4	
Adenopathy	2	
Abscess	1	

^1^ BPH: benign prostatic hyperplasia; ^2^ Foreign bodies were located in the subcutaneous/muscular tissues, gastro-jejunal tract, pulmonary caudal lobe, and peritoneal cavity.

**Table 9:** Typologies of the incidentalomas in the CT examinations in dogs and cats based on the age of the patients. When there was more than one, the number is reported in brackets.

Age (Years)	Typologies of Incidentalomas
<1	Bronchopneumonia; airway collapse
1	Sialocele
2	Hiatal hernia; facet joint hypertrophy
3	(None)
4	Hiatal hernia
5	Herniated disc (2); mandibular cyst; foreign body; foraminal stenosis; facet joint hypertrophy; otitis media; splenic nodule
6	Spondylopathy (2); intracanal fat accumulation; herniated disc (2); congenital/traumatic spinal deviation; otitis media
7	Spondylopathy; herniated disc; pituitary volume increase; sialocele; hiatal hernia; otitis media
8	Foraminal stenosis; herniated disc (3); otolithiasis; otitis media
9	Pulmonary thromboembolism; peritoneal nodule; splenic nodule (2); adrenal neoplasm; foreign body, herniated disc (2)
10	Herniated disc (2); renal lithiasis/dystrophic mineralization (2); hiatal hernia; thyroid nodule; pancreatic volume increase and peri-pancreatic steatitis; adenopathy
11	Herniated disc (2); cholelithiasis; adrenal neoplasm; otitis externa
12	Foraminal stenosis; prostatic cyst/BPH (4); patellar luxation; adenopathy; bronchopneumonia; abscess; foreign body (2); herniated disc; cholelithiasis; adrenal hyperplasia
13	Herniated disc (2); prostatic cyst/BPH
14	Foraminal stenosis; lung thickening; splenic nodule
15	Airway collapse (2); renal lithiasis/dystrophic mineralization; otic polyp; urolithiasis; thyroid nodule.

## Data Availability

The data presented in this study are not publicly available due to privacy restrictions.
